# Impact of the Cusa and Operative Ultrasound on Hepatic Resection

**DOI:** 10.1155/1991/62986

**Published:** 1991

**Authors:** J. M. Little, M. J. Hollands

**Affiliations:** 1 Department of Surgery Westmead Hospital, and The University of Sydney Australia; 2 Department of Surgery Westmead Hospital Westmead N.S.W. 2145 Australia

## Abstract

New technologies have been developed for liver surgery, and, like all new technologies, they have a glamour which makes them seem desirable. There is an understanding abroad that they make liver surgery easier and open up the field to those without special training. But there is no proof that the new devices are in any way cost-effective, and certainly no proof that liver surgery has become safer since their advent. Fifty consecutive elective liver resections have been studied, almost half performed with the aid of the ultrasonic dissector and aspirator and diagnostic intraoperative ultrasound. There was no mortality in the whole group, but a 24% morbidity. Operative diagnostic ultrasound was thought to allow more precise planning of surgery. Its use was not associated with any increase in operative time, nor was there any increase in postoperative morbidity. The ultrasonic dissector and aspirator improved technique, reflected in a lower blood loss for each case, in fewer transfusions required, in a shorter postoperative hospital stay and in an ability to achieve these benefits in older patients. Neither device could be said to offer an entree to instant liver surgery. The use of the two devices apparently offered savings measured by a fall in the median postoperative hospital stay of 4.5 days, by a saving of 700 mls in median blood requirement and by a fall in transfusion rate from 64% to 9%.


*HPB Surgery*, 1991, Vol. 3, pp. 271–278
Reprints available directly from the publisher
Photocopying permitted by license only

© 1991 Harwood Academic Publishers GmbH
Printed in the United Kingdom

### IMPACT OF THE CUSA AND OPERATIVE ULTRASOUND ON HEPATIC RESECTION

J.M. LITTLE and M.J. HOLLANDS
*Department of Surgery, Westmead Hospital, and The University of Sydney*

*(Received 29 August 1990)*

New technologies have been developed for liver surgery, and, like all new technologies, they have a glamour which makes them seem desirable. There is an understanding abroad that they make liver surgery easier and open up the field to those without special training. But there is no proof that the new devices are in any way cost-effective, and certainly no proof that liver surgery has become safer since their advent. Fifty consecutive elective liver resections have been studied, almost half performed with the aid of the ultrasonic dissector and aspirator and diagnostic intraoperative ultrasound. There was no mortality in the whole group, but a 24% morbidity. Operative diagnostic ultrasound was thought to allow more precise planning of surgery. Its use was not associated with any increase in operative time, nor was there any increase in postoperative morbidity. The ultrasonic dissector and aspirator improved technique, reflected in a lower blood loss for each case, in fewer transfusions required, in a shorter postoperative hospital stay and in an ability to achieve these benefits in older patients. Neither device could be said to offer an entree to instant liver surgery. The use of the two devices apparently offered savings measured by a fall in the median postoperative hospital stay of 4.5 days, by a saving of 700 mls in median blood requirement and by a fall in transfusion rate from 64% to 9%.

KEY WORDS: Hepatic resection, ultrasonic aspiration dissector (CUSA), operative ultrasound, operative morbidity

### INTRODUCTION

The techniques of modern hepatic surgery were defined in the early 1950's[1,2], and have changed little since then. A recent surge of technical development has attracted a great deal of attention. The operative ultrasound machine[3–7] appears in more operating theatres, and surgeons have written of its diagnostic virtues. New devices for dissecting liver parenchyma have appeared — the ultrasonic aspiration dissector (CUSA)[8–11], the water jet[12], the laser[9] and the microwave coagulator[13] are examples. New haemostatic agents — including lyophilised collagen and fibrin glue[14,15] — are available in some parts of the world. Reports of the use of these new devices and materials are generally enthusiastic.

There are, however, voices of conservatism. Adson[16] has pointed out that he operates well without these aids, and questions whether they are as attractive as some have claimed. Questions about the long term benefits that may come from more accurate staging of tumours with diagnostic ultrasound will not be answered for years to come. Questions about the short term benefits, on the other hand,

Address correspondence to: Professor J.M. Little, Department of Surgery, Westmead Hospital, Westmead N.S.W. 2145, AUSTRALIA.

should be answerable by now, and there are already some suggestions that blood loss is less with the CUSA[10,11]. The present paper offers an evaluation of the place of operative ultrasound and of the CUSA in elective liver surgery.

### MATERIALS AND METHODS

Fifty patients have been studied, having had elective hepatic resections between January 1984 and July 1989. No selection criteria have been applied, except that the operations were truly elective.

The principles of the operative technique were standardised. Tumours were carefully staged by laparotomy, palpation and inspection. In the latter part of the study, operative ultrasound was used routinely as an adjunct. The liver was extensively mobilised, and the porta hepatis was dissected. The extent of resection was mapped, and the porta hepatis cross clamped with a peripheral vascular clamp. The times of clamping and release were noted. Parenchymal transection was then undertaken with a blunt instrument. Latterly, the CUSA was used for this purpose. The hepatic and portal vessels and ducts were displayed within the parenchyma, and controlled with metal clips, ligatures and sutures. Gelatin foam soaked in topical thrombin was applied to the cut surface when the resection was completed. When any residual bleeding or bile leakage had been controlled, one or two closed suction drains were inserted and left until their drainage volume was less than 30 mls/day. Patients were nursed for three to four days postoperatively in a high dependency ward. Intensive care beds were not used. Parenteral or enteral nutrition were used if indicated.

Data were maintained, using a data base written in dBase III, by JML in the Department of Surgery at this hospital. The usual identifying details were entered. Operation date was recorded. The level of operation was recorded as the number of anatomic segments removed. For this purpose, segment IV was regarded as two segments — the anterior and posterior portions, or segments IVa and IVb. The total time of portal clamping was recorded. So were operation time and total measured blood loss. A note was also made of whether transfusion of blood was required. It was also noted whether operative ultrasound and the CUSA were used.

Significant morbidity was defined as a postoperative complication requiring a significant modification of treatment, radiological or surgical intervention or prolonged stay in hospital. The following were regarded as part of the "normal" course in hospital: fever not ascribed to a specific infective episode and not requiring specific treatment, lung collapse responding to physiotherapy and a short course of antibiotics, temporary hypoalbuminaemia not requiring nutritional support, a small perihepatic collection not requiring drainage. The hospital stay from the day of operation was also calculated.

Non parametric statistical methods (Wilcoxon rank sum, chi square, Fisher's exact test) were used to compare the characteristics of those patients operated with the CUSA and the operative ultrasound to those of patients operated using earlier techniques. Multivariate techniques (multiple regression, principal component analysis) were used to examine the determinants of morbidity and of good risk.

### RESULTS

### *General*

There were 50 patients in the series, 23 men and 27 women. Their median age was 51 years (range 11–70 years). Twenty four had colonic secondaries, 7 hepatocellular carcinomas, 6 intrahepatic stones, 5 hydatids, 4 areas of focal nodular hyperplasia and one each a bile duct adenoma, a cholangiocarcinoma, a cystadenoma and a leiomyoma.

The accrual of patients has tended to increase with the passing years (Table 1), with 6 operations performed in 1984 and 12 in the first half of 1989. Operation levels are shown in Table 2.

**Table 1**

| | |
|---|---|
| 1984 | 6 |
| 1985 | 3 |
| 1986 | 8 |
| 1987 | 11 |
| 1988 | 10 |
| 1989 (first 6 months) | 12 |

**Table 2**

| | |
|---|---|
| 1 segment | 10 |
| 2 segments | 19 |
| 3 segments | 4 |
| 4 segments | 14 |
| 5 segments | 1 |
| 6 segments | 2 |

### *Portal clamping times*

The median time of portal clamping was 15 minutes, with a range of 5 to 35 minutes.

### *Operation times*

The median time from the start of the surgical part of the procedure to the time the patient left the theatre was 181 minutes, with a range of 90 to 400 minutes.

### *Blood loss*

The median blood loss was 450 mls, with a range of 100 to 4500 mls. Twenty patients were given blood transfusion during or after operation.

### *Operative ultrasound*

The diagnostic ultrasound was used 23 times in the 50 patients.

### *CUSA*

The CUSA, which first became available in 1987, was used 22 times. It was not available for all patients during the period from 1987 to 1989, so that it has been possible to distinguish between the impact of the device and the benefits of experience.

### *Differences between CUSA and non-CUSA groups*

Patients in whom the CUSA was used were significantly older than those operated before the device became available. The median age of the CUSA group was 59 years (range 24–70 years), compared to 44.5 years (range 11–69) for the non-CUSA group. Wilcoxon testing showed this difference to be significant, $p = .0063$.

Portal clamping times were longer in the CUSA group (median 18.5 minutes, range 8–35 minutes), compared with 13 minutes, range 5–35 minutes ($p = .031$, Wilcoxon test).

Blood loss was less in the CUSA group — 275 mls (100–850 mls), compared with 950 mls (150–4500 mls) in the non-CUSA group. This difference was significant on Wilcoxon testing, $p = .0039$. Transfusions were less frequently used in the CUSA group — 2 of 22 patients, compared with 18 of 28 in the non-CUSA group, $p = .0001$, Fisher test.

The CUSA group had a shorter median hospital stay — 9.5 days (8–23 days), compared with 14 days (9–42 days) for the non-CUSA group, $p = .0041$, Wilcoxon test.

No other significant differences were noted. In particular, there were no differences in operating times nor in the incidence of major morbidity.

### *Influence of operative ultrasound*

The use of operative ultrasound did not increase operating times. The median operating time for the ultrasound group was 168 minutes (range 105–340 minutes), while that for the non-ultrasound group was 185 minutes (range 90–400 minutes). This difference was not significant ($p = .1103$, Wilcoxon test).

Nor was there any difference in the incidence of major complications between the two groups. Five of 23 of those in the ultrasound group suffered major complications, compared with 8 of 27 in the non-ultrasound group ($p = .76$, chi square).

### *Independent determinants of morbidity*

There were six factors which emerged as determinants of significant morbidity on multiple regression analysis — 1. prolonged operating times, 2. higher blood loss, 3. blood transfusion, 4. female sex, 5. advancing age, and 6. not using the CUSA.

Principal components analysis suggested two factors that might determine a straightforward postoperative course. The first was the experience of the operator (morbidity has declined with the passing years) together with the use of the CUSA and the operative ultrasound. The second was the use of the least possible operation, removing the least liver and involving the shortest portal clamp times, the shortest operating time and the least blood loss.

### DISCUSSION

The new technologies for liver surgery are expensive, but are they cost-effective? When something new comes on the market, there is a quite natural feeling that everyone should use it if they are to remain competitive. Possession of the new becomes an essential part of continuing surgical credibility, and thoughtful evaluation seems less important. This syndrome has affected the acceptance of the CUSA and the operative ultrasound into the practice of liver surgery. Yet the techniques of liver surgery have been well established since the early 1950's[1,2], and it is well documented that the mortality of liver resection in the absence of cirrhosis is less than 5%[17–19], although the morbidity remains high. The long term benefits of resection for tumors are also clearly established[17–20]. In order to justify expensive new equipment, then someone will have to demonstrate savings in cost, time or suffering, or show that long term results are improved or that safe liver surgery is made available to more people. It will be years before the long term survival of those with hepatic malignancy can be reassessed, and years before we can judge whether liver surgery has been opened up to more of those with surgically treatable liver lesions. The present study has demonstrated that significantly older patients have been coming to surgery since the advent of operative ultrasound and the CUSA, but there may be other explanations for this observation, including a broadening referral base.

It will be a long time before we know whether operative ultrasound is really helping us to stage liver cancer more accurately. What can be said so far is that the technique is not easy to learn and that the device is not infallible in picking up small lesions in the parenchyma. The process of adjusting gain to suit the various probes is important, and not every imaging department can spare a technician to help when the surgeon is working. Theatre staff must learn to maintain and adjust the machine, as well as to care for and sterilise the probes. The learning process takes time and patience. The present study does suggest that there are no increases in operating times with its use, nor any associated morbidity. The lack of prolongation of operating time is a little surprising, since it takes 10 to 15 minutes to complete the ultrasonic examination of the liver. It is possible that the improved planning that results from the ultrasonic information actually saves time at the next step of the resection. It remains to be seen whether improved staging will lead to better long term results with better patient selection for resection, and whether it becomes possible to carry out more limited resections for tumours shown to be ultrasonically localised. The present study has not shown any significant change in the extent of the resections carried out before and after the availability of the ultrasound machine.

It can equally be said that the CUSA does not make an instant liver surgeon. In fact, the reverse is true. The ability to dissect in a controlled fashion within the parenchyma of the liver and to perform segmental resections makes a detailed, working knowledge of intrahepatic anatomy absolutely essential. It is easy to disconnect segments of liver from blood supply and venous or biliary drainage unless the surgeon knows very well what he is encountering in the depths of the liver.

The CUSA does allow a drier operating field and excellent operating conditions. These advantages are demonstrated by a lower blood loss among the

CUSA-treated patients and a lower transfusion rate, an important consideration in the present era of concern about HIV and hepatitis B transmission and in the face of periodic shortages of blood for transfusion. Multivariate analysis demonstrates that this economy of blood usage is associated with the CUSA and not with the operation year. It appears, therefore, to depend on the technology rather than on increasing expertise. We have been impressed by the dryness of the field when the resection is finished and by the relatively straightforward postoperative course experienced by CUSA-managed patients. Their median postoperative stay in hospital has been shorter by some 4.5 days, a saving in patient and hospital expenses. These advantages are secured at the expense of longer portal clamp-times. Fortunately, these have not been associated with any observable morbidity. Total operating times have not increased.

Although both the CUSA and the operative ultrasound do seem to offer advantages, there is no argument for making them both available in every hospital. Surgeons manage most things that they do without their help, and both are expensive. Both require continuing use for skills to be developed and maintained. Both are valuable adjuncts to surgery in busy, specialised units with a major interest in hepatic surgery. Both pieces of equipment can be shared — with neurosurgeons, urologists, cardiac and vascular surgeons, orthopaedic surgeons and gastroenterologists, all of whom will find uses for one or both devices. Both represent progress in surgery. Neither can substitute for basic training in anatomy, pathology and surgical technique.

### *References*

1. Lortat-Jacob, J.L. and Robert, H.G. (1952) Hepatectomie droit reglee. *Presse med*, **60**, 549–551
2. Quattlebaum, J.K. (1953) Massive resection of the liver. *Ann. Surg.*, **137**, 787–795
3. Castaing, D., Kunstlinger, F., Habib, N. and Bismuth, H. (1985) Intraoperative ultrasonographic study of the liver. Methods and anatomic results. *Amer. J. Surg.*, **149**, 676–682
4. Bismuth, H. and Castaing, D. (1987) Operative ultrasound of the liver and biliary ducts pp. 1–91. Springer Verlag: Berlin, Heidelberg, New York, London, Paris, Tokyo
5. Bismuth, H., Castaing, D. and Garden, O.J. (1987) The use of operative ultrasound in surgery of primary liver tumours. *World. J. Surg.*, **11**, 610–614
6. Makuuchi, M., Hasegawa, H., Yamazaki, S., Takayasu, K. and Moriyama, N. (1987) The use of operative ultrasound as an aid to liver resection in patients with hepatocellular carcinoma. *World. J. Surg*, **11**, 615–621
7. Boldrin, G., de Gaetano, A.M., Giovannini, I., Catagneto, M., Colagrande, C. and Castiglioni, G. (1987) The systematic use of operative ultrasound for detection of liver metastases during colorectal surgery. *World J. Surg.*, **11**, 622–627
8. Hodgson, W.J.B and DelGuerico, L.R.M. (1984) Preliminary experience in liver surgery using the ultrasonic scalpel. *Surgery*, **95**, 230–234
9. Tranberg, K-G, Rigotti, P., Brackett, K.A., Bjornson, H.S., Fischer, J.E. and Joffe, S.N. (1986) Liver resection: a comparison using the Nd-YAG laser, an ultrasonic surgical aspirator, or blunt dissection. *Amer. J. Surg.*, **151**, 368–373
10. Andrus, C.H., and Kaminski, D.L. (1986) Segmental hepatic resection utilizing the ultrasonic dissector. *Arch. Surg.*, **121**, 515–521
11. Hodgson, W.J.B., Kemeny, M., Scheele, J. and Tranberg, K-G (1987) Does the ultrasound dissector improve the quality of hepato-pancreatico-biliary surgery? In *Progress in surgery of the liver, pancreas and biliary system,* pp 143–162, ed. S. Bengmark. Dordrecht, Boston, Lancaster: Martinus Nijhoff
12. Papachristou, D.N. and Barters, R. (1982) Resection of the liver with a water jet. *Brit. J. Surg.*, **69**, 93–94
13. Tabuse, K., Katsumi, M., Kobayashi, Y., Noguchi, H., Egawa, H. *et al.* (1985) Microwave surgery: hepatectomy using a microwave tissue coagulator. *World J. Surg.*, **9**, 136–143

14. Giakoustidis, E., Drosinopoulos, P. Agouridakis, K., Galanis, N. (1985) Surgical treatment of liver injuries by application of fibrinkleber. *World. J. Surg.*, **9**, 144–148
15. Teboul, F., Peix, J.L., Berard, P., Gouillat, C., Faysse, E., Viguier, M. and Saubier, E. (1987) Interet de la colle de fibrine apres chirurgie d'exerese hepatique. *Lyon Chir.*, **83**, 60–62
16. Adson, M.A. (1986) In Discussion of Andrus, C.H., Kaminski, D.L. Segmental hepatic resection utilizing the ultrasonic dissector. *Arch. Surg.*, **121**, 520–521
17. Adson, M.A., van Heerden, J.A. and Adson, M.H. (1984) Resection of hepatic metastases from colorectal cancer. *Arch. Surg.*, **119**, 647–651
18. Little, J.M., and Hollands, M.J. (1987) Hepatic resection for colorectal metastases. Selection of cases and determinants of success. *Aust. NZ. J. Surg.*, **57**, 355–359
19. Hughes, K.S., Simon, R., Songhorabodi, S. *et al.* (1988) Resection of the liver for colorectal metastases: a multi-institutional study of indications for resection. *Surgery*, **103**, 278–288
20. Adson, M.A. (1988) Primary cancer of the liver: considerations about resection, in Progress in surgery of the liver, pancreas and biliary system, pp. 179–187, ed. S. Bengmark, Dordrecht, Boston, Lancaster: Martinus Nijhoff

*(Accepted by S. Bengmark 29 August 1990)*

### INVITED COMMENTARY

Since its first application in liver surgery by Makuuchi and Hasegawa in Japan at the beginning of the 80's, intraoperative ultrasound has become increasingly widely used in hepatic surgery centers in the West. Intraoperative ultrasound, which our unit started using in 1984[1], is now routinely used in operations for both neoplastic and benign lesions, just as cholangiography is practised in biliary surgery. As with cholangiography, surgeons themselves are now able to carry out the investigation and interpret the ultrasound images. A training period alongside ultrasound specialists is indispensable and is perhaps the main drawback to wider use of this technique. In fact the surgeon must himself perform the ultrasonography if the operation is to be truly echo-guided and help avoid accidental damage of intrahepatic structures and enable more radical tumor ablation by pinpointing a resection line at a safe distance from the lesion. In skilled hands routine ultrasound exploration does not lengthen operation time by more than 10 minutes.

Intraoperative ultrasound affords "real time" investigation during surgery of the layout of the intrahepatic vessels, displaying any anatomical anomalies and showing intrahepatic vascular involvement in neoplastic or benign diseases. It provides a comprehensive segmental map for both normal as well as cirrhotic or otherwise damaged livers where structures have undergone alteration on account of the mass growth or previous operations. Moreover intraoperative ultrasound can pick up intrahepatic lesions as small as 4–5 millimeters which would not be detected by CT scan, angiography or preoperative ultrasound. This is because intraoperative probes have a higher sound frequency than conventional abdominal transducers (5 or 7.5 or even 10 MHz), and therefore resolution is increased.

A prospective study was conducted in my Institution in 54 patients undergoing surgery for primary liver tumors (32 cases) or metastases (22 cases)[2]. Most cases were asymptomatic and initial diagnoses were made on the basis of a strict echographic follow-up. All patients underwent angiography, CT scan and echography using 3.5 MHz probes. The findings were compared with the results of intraoperative ultrasound (5 MHz probes), surgical exploration of the liver and the pathological examination of the specimens in 37 patients who underwent a liver resection. The sensitivity of intraoperative ultrasonography was 95.4% in non cirrhotic patients and 91.1% in cirrhotics; preoperative ultrasonography had a sensibility of 84.1% and 73.5% respectively; CT scan 77.2% and 17%; angiography 65.9% and 58.8%. Intraoperative ultrasound proved of greater diagnostic value especially in the case of minute lesions, smaller than 1 cm, which often escape pre-operative examinations. The most rewarding results were in the cirrhotic group where a considerable percentage of the lesions found were not palpable during laparotomy.

Another study has been carried out on the ability of intraoperative ultrasound to detect occult synchronous liver metastases in patients with digestive tumors. 110 patients operated on for gastrointestinal or pancreatic tumors were prospectively investigated. All patients had preoperative ultrasound and careful palpation of the liver at surgery. Occult liver metastases were demonstrated by intraoperative ultrasound in 8 cases; 3 patients operated for a carcinoma of the head of the pancreas (in all cases the pancreatic resection planned was not carried out), 5 with a rectal carcinoma and 1 with a gastric cancer. The majority of these lesions were 1 cm or less, but in 2 patients metastases were 3 and 4 cm respectively: the lesions were hidden in the 7th segment in the "blind area" of the abdominal echographic exploration and difficult to palpate being covered by the insertion of the triangular ligament.

Intraoperative ultrasonography is the ultimate diagnostic examination to detect liver tumors and as such can prompt more radical surgery where this is feasible, while excluding patients with disseminated intrahepatic disease who would derive no lasting benefits from surgery. The advantages of this technique are expecially conspicuous in the case of cirrhotic livers where surgical exploration is more difficult and parenchimal tumors often fail to be detected with palpation[3].

Even if, as Little notes in his article, many colleagues have and continue to perform brilliant liver surgery using the conventional approach, I believe, in the light of more than 6 years experience with intraoperative ultrasound, that this technique provides great advantages especially for more radical surgery, detecting small non-palpable lesions, allowing for more accurate segment resections and altogether making ablative surgery more rational by avoiding resection in cases where intrahepatic disease is too disseminated to make surgery worthwhile.

### *References*

1. Gozzetti, G., Mazziotti, A. and Coll. (1986) Intraoperative ultrasonography in surgery for liver tumors. *Surgery*, **99**, 573--579
2. Gozzetti, G., Mazziotti, A. and Coll. (1989) Intraoperative ultrasonography in hepato-biliary and pancreatic surgery. Kluwer Acad. Publ.
3. Gozzetti, G., Mazziotti, A. and Coll. (1988) Clinical experience with liver resections for hepatocellular carcinoma in patients with cirrhosis. *Surg. Gynecol. Obstetr.*, **166**, 503–510

Giuseppe Gozzetti
Bologna, Italy

